# Bioimaging marine crustacean brain: quantitative comparison of micro-CT preparations in an Alpheid snapping shrimp

**DOI:** 10.3389/fnins.2024.1428825

**Published:** 2024-11-26

**Authors:** Lucille Chapuis, Cara-Sophia Andres, Dane A. Gerneke, Craig A. Radford

**Affiliations:** ^1^Leigh Marine Laboratory, Institute of Marine Science, University of Auckland, Leigh, New Zealand; ^2^Bioengineering Institute, University of Auckland, Auckland, New Zealand

**Keywords:** micro-CT, nervous system, *Alpheus richardsoni*, 3D visualization, non-invasive imaging

## Abstract

Non-invasive bioimaging techniques like X-ray micro-computed tomography (μCT), combined with contrast-enhancing techniques, allow the 3D visualization of the central nervous system *in situ*, without the destruction of the sample. However, quantitative comparisons of the most common fixation and contrast-enhancing protocols are rare, especially in marine invertebrates. Using the snapping shrimp (*Alpheus richardsoni*) as a model, we test three common fixation and staining agents combinations to prepare specimens prior to μCT scanning. The contrast ratios of the resulting images are then quantitatively compared. Our results show that a buffered iodine solution on a specimen fixed with 10% formalin offers the best nervous tissue discriminability. This optimal combination allows a semi-automated segmentation of the central nervous system organs from the μCT images. We thus provide general guidance for μCT applications, particularly suitable for marine crustaceans. Species-specific morphological adaptations can then be characterized and studied in the context of evolution and behavioral ecology.

## 1 Introduction

The central nervous system (CNS) of marine invertebrates is relatively unexplored, notably due to the difficulty of properly preserving and observing these organs *in situ*. While classical techniques in histology and immunohistochemistry combined with light and electron microscopy have been and are still fundamental into acquiring detailed neuroanatomical information, they are often prone to artifacts due to compression and distortion of the tissues when sectioned. They also are invasive procedures often resulting in the destruction of the specimen. Non-invasive bioimaging, such as X-ray micro-computed tomography (μCT), overcomes some of these limitations and has been shown valuable for visualizing and quantifying internal anatomy and structural complexity in invertebrates for both morphological and ecological studies (e.g., Sombke et al., 2015; Gutiérrez et al., 2018).

Hard structures (e.g., shells, skeletons, mouth parts of invertebrates) are primarily imaged in μCT due to the large difference in X-ray attenuation of mineralized tissues. In order to extend the use of this technique to the visualization of soft tissues, such as that of the nervous system, the specimen can be stained prior to imaging using contrast-enhancing agents. In marine invertebrates, staining agents like iodine, phosphotungstic acid or osmium tetroxide have been successfully applied to soft tissues of different species, for example in cnidarians (Holst et al., 2016; Gusmo et al., 2018), polychaetes (Dinley et al., 2010; Faulwetter et al., 2013b), solenogastres mollusks (Martínez-Sanjuán et al., 2022), cephalopods (Kerbl et al., 2013; Sakurai and Ikeda, 2019), echinoderms (Stoehr et al., 2019; Ziegler, 2019), gastropods (Sumner-Rooney et al., 2019), bivalves (Machado et al., 2018), and platyhelminthes (Ikenaga et al., 2024). Contrast-enhancing techniques also sometimes include the use of a drying step before scanning (Sombke et al., 2015; Krieger and Spitzner, 2020), although this has been shown to induce shrinkage and also alters the tissues indefinitely (Faulwetter et al., 2013a; Sombke et al., 2015; Holst et al., 2021).

The versatility of μCT for the visualization of internal organs and tissues has rarely been tested and confirmed in marine crustaceans. Crustaceans often present some challenges, containing both low density soft tissues (e.g., CNS) and very dense calicified structures (e.g., carapace) which need to be visualized. The hydrothermal vent shrimp *Rimicaris exoculata* (Machon et al., 2019), larval crabs *Carcinus maenas* (Krieger and Spitzner, 2020), and the amphipod *Parhyale hawaiensis* (Wittfoth et al., 2019) were all stained with alcoholic iodine and critical point dried prior to μCT scanning in order to investigate their brain. Critical point drying was also used before scanning the Pacific white shrimp *Penaeus vannamei* (Meth et al., 2017). Finally, Nischik and Krieger (2018) used alcoholic iodine to stain and scan juveniles of the marbled crayfish *Procambarus fallax* forma *virginalis* to study the effect of sample preparation on the volumetric preservation of the nervous system. While these studies all successfully imaged some crustacean CNS structures, a quantitative comparison between different protocols in order to identify optimal fixation and staining agents is lacking.

Here, a time-efficient, low-cost, non-invasive procedure for the optimal visualization of the crustacean CNS was investigated, using the snapping shrimp, *Alpheus richardsoni*, as a model. Alpheid shrimps represent one of the most diverse groups of marine decapod crustaceans, abundant in tropical and subtropical shallow water habitats, and offer opportunities to study the evolution (i.e., including the neuroanatomical basis) of intriguing behaviors like spectacular claw snapping, facultative and obligate symbioses with many animal groups, and eusociality (Anker et al., 2006).

Three simple and common fixation (formalin and ethanol) and staining protocols (water-based and alcoholic iodine) were tested, including different staining durations, to enhance the contrast of the soft tissues. Following reconstruction of the μCT images, contrast ratio were quantitatively compared between the identified CNS organs for each combination and staining duration. Standardized scans were then used to formally juxtapose the three procedures. Finally, the CNS organs from an optimal scan are segmented and volumetrically measured, thus confirm the application of the best fixation and staining methodology for identifying and reconstructing the CNS in a marine crustacean.

## 2 Methods

Three fixation and staining protocols were tested: (i) formalin 10% + Sorenson’s buffered water-based iodine (B-Lugol 1.25%), (ii) ethanol 95% + Sorenson’s buffered water-based iodine (B-Lugol 1.25%), and (iii) ethanol 95% + alcoholic iodine (I_2_E 1%). Formalin 10% and ethanol are two very popular fixative agents used to fix invertebrate tissues (Williams and Syoc, 2007). Similarly, as mentioned above, iodine has been shown as an effective staining compound for both vertebrate and invertebrate soft tissues (Metscher, 2009a) and can be both used in combination with distilled water and ethanol. Because significant tissue shrinkage can be induced by iodine, we chose to use a buffered iodine solution to minimize the effect in our samples (Dawood et al., 2021). Note that fixation with formalin 10% followed by staining with alcoholic iodine (I2E 1%) was not investigated, as assumed less effective than directly staining with water-based iodine (considering the results presented below) and avoiding a stepwise transfer from formalin to ethanol.

### 2.1 Sample preparation and scanning

Five snapping shrimps (*Alpheus richardsoni*) of average total length ± standard deviation 22.8 ± 3.8 mm were collected from mudflats in Omaha, Auckland, New Zealand in March–April 2023. The shrimps were euthanized in an ice slurry and directly immersed in either phosphate buffered saline (PBS)10% formalin for minimum 24 h or ethanol (EtOH) 95% for minimum 48 h (tissue to solution 1:9 volume ratio). They were then stained with either a buffered water-based iodine solution (B-Lugol 1.25%) or alcoholic iodine (1% I_2_ in 100% EtOH) during 0, 12, 24, 48, 72, and 96 h as summarized in Table 1 (specimen ID AR01 – AR05). Each shrimp was immersed in approximately nine times its volume of staining solution in opaque sample tubes. The staining solutions were visually monitored to avoid any iodine depletion (i.e., staining solution becoming clearer), but none of the solutions needed refreshing before the 96 h mark. The specimen AR05 (fixed in EtOH, but stained with B-Lugol) was first downgraded stepwisely every 48 h from 95, to 70%, 50 and 30% EtOH before being transferred in the B-Lugol 1.25% solution. Before scanning, the specimens were rinsed in Sorenson’s buffer or EtOH (100%), respective to the staining agent. They were placed in a plastic tube, in air, and held in place with polystyrene pads. Micro-CT was performed with a Bruker Skyscan 1,172 (Bruker, Kontich, Belgium) at the Auckland Bioengineering Institute; parameters for each scan are presented in Table 1 (scan ID 01–22).

**Table 1:** Details of the fixation and staining agents used for each specimen *Alpheus richardsoni* and X-ray scanning parameters for each scan.

Scan ID	Specimen ID	Fixation	Staining agent	Staining time (hours)	Current (μA)	Voltage (kV)	Exposure time (ms)	Pixel size (μm)	Filter
01	AR01	Formalin 10%	B-Lugol 1.25%	0	134	75	1,000	2.1	No filter
02	AR01	Formalin 10%	B-Lugol 1.25%	12	100	100	1,200	2.1	No filter
03	AR01	Formalin 10%	B-Lugol 1.25%	24	100	100	1,200	2.1	No filter
04	AR01	Formalin 10%	B-Lugol 1.25%	48	100	100	1,600	2.1	No filter
05	AR01	Formalin 10%	B-Lugol 1.25%	72	100	100	1,500	2.1	No filter
06	AR02	Formalin 10%	B-Lugol 1.25%	0	152	66	1,000	2.7	No filter
07	AR02	Formalin 10%	B-Lugol 1.25%	24	100	100	800	2.7	No filter
08	AR02	Formalin 10%	B-Lugol 1.25%	48	100	100	1,000	2.7	No filter
09	AR02	Formalin 10%	B-Lugol 1.25%	72	100	100	1,000	2.7	No filter
10	AR03	Formalin 10%	B-Lugol 1.25%	0	110	69	1,000	2.1	No filter
11	AR03	Formalin 10%	B-Lugol 1.25%	24	100	100	1,100	2.1	No filter
12	AR03	Formalin 10%	B-Lugol 1.25%	48	100	100	1,100	2.1	No filter
13	AR03	Formalin 10%	B-Lugol 1.25%	72	100	100	1,200	2.1	No filter
14	AR03	Formalin 10%	B-Lugol 1.25%	96	100	100	1,000	2.1	No filter
15	AR04	EtOH 95%	I_2_E 1%	0	100	75	1,000	2.5	No filter
16	AR04	EtOH 95%	I_2_E 1%	12	110	91	1,100	2.5	No filter
17	AR04	EtOH 95%	I_2_E 1%	24	110	91	1,100	2.5	No filter
18	AR04	EtOH 95%	I_2_E 1%	48	100	100	1,100	2.5	No filter
19	AR05	EtOH 95%	B-Lugol 1.25%	0	134	75	1,000	2.1	No filter
20	AR05	EtOH 95%	B-Lugol 1.25%	12	100	100	1,200	2.1	No filter
21	AR05	EtOH 95%	B-Lugol 1.25%	24	100	100	1,200	2.1	No filter
22	AR05	EtOH 95%	B-Lugol 1.25%	48	100	100	1,600	2.1	No filter
23	AR05	EtOH 95%	B-Lugol 1.25%	72	100	100	1,500	2.1	No filter
24	AR06	Formalin 10%	B-Lugol 1.25%	48	100	100	700	3.0	2xBUILD^*^
25	AR07	EtOH 95%	I_2_EtOH	48	100	100	700	3.0	2xBUILD^*^
26	AR08	EtOH 95%	B-Lugol 1.25%	48	100	100	700	3.0	2xBUILD^*^

* 2xBUILD filter was made of two layers of building Aluminum tape (3M Scotch Foil Tape 3,311, St. Paul, MN, United States) on 0.13 mm styrene sheet support.

### 2.2 Reconstruction and contrast ratio

The images were reconstructed using Nrecon 2.2.2 software from Bruker and outputted as 16-bit grayscale values .tif files (i.e., pixel value ranging from 0 to 65,535). The same reconstructions parameters were used for all scans: smoothing = 3, ring artifact reduction = 26, beam hardening correction = 66, minimum contrast limit = 0, maximum contrast limit = 20% higher than the maximum attenuation of the material of principal interest for reconstruction. Only the misalignment compensation was tuned differently for each scan.

**Figure 1:**
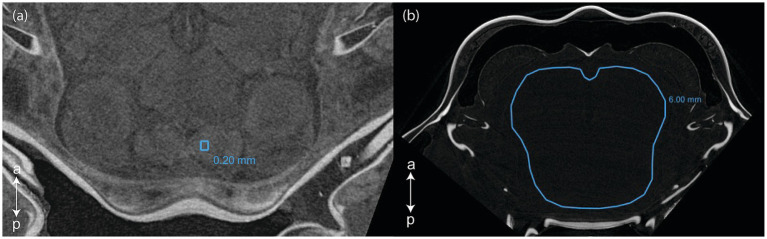
Selections of (a) a 0.2 mm circumference area in the right lateral antennular neuropil and (b) a 6.0 mm circumference area in a poorly contrasted scan (Dragonfly, 2022). a, anterior; p, posterior.

Scans were then visualized and analyzed using Dragonfly (2022) software version 2022.2. Patches of 0.20 mm circumference were selected in a homogenous section of each of the following selected CNS organ: lamina, external medulla, lobula, hemiellipsoid body neuropils, anterior medial protocerebral neuropil, posterior medial protocerebral neuropil, olfactory lobe, lateral antennular neuropil, antenna II neuropil (Figure 1a). Labeling of the CNS structures was done by topological correspondence to the neuroanatomical descriptions of other crustaceans (Sandeman et al., 1992, 1993; Mellon, 2014; Meth et al., 2017; Machon et al., 2019, 2020; Krieger et al., 2020; Lin et al., 2021). The gray values of the patches were sampled for both the right and the left organs, and then averaged. For specimen with very poor contrast, the gray values were taken in a 6.00 mm circumference area where the organs would have been located (although not distinguishable) (Figure 1b). Two background values were taken from two 0.20 mm circumference areas located near the center of the tube enclosing the specimen. For all selected surfaces the mean and standard deviation gray values were recorded from the histogram.

The contrast ratio (*R*) was determined as the difference in mean gray value (*μ*) between patches of background and CNS organs, divided by the mean background gray value:


(1) $$R=\frac{\mu_{\mathrm{organ}}-\mu_{\mathrm{background}}}{\mu_{\mathrm{background}}}$$

R statistical software (V 4.3.0, R Core Team, 2021) was used to plot the contrast ratio differences for each staining protocol and Kruskal–Wallis and Dunn (1964) multiple comparisons tests adjusted with the Benjamini–Hochberg method were used to indicate significance differences, using R packages ggplot2 (Wickham, 2016) and FSA (Ogle et al., 2023).

Finally, as a way to investigate the discriminability of each CNS organ between them (and irrespective of the background), an ‘inter-organ’ (Equation 2) contrast ratio (*R_inter_*) was calculated as the difference between the highest and the lowest gray value (*μ*) among all identified CNS organs, divided by the lowest gray value:


(2) $$R_{\mathrm{inter}}=\frac{\max\left(\mu_{\mathrm{organ}}\right)-\min\left(\mu_{\mathrm{organ}}\right)}{\min\left(\mu_{\mathrm{organ}}\right)}$$

### 2.3 Standardized scans

Three extra *A. richardsoni* specimens (specimen ID AR06–08) were caught from the same location as above (Omaha mudflats) in January 2024, and were stained for 48 h with the three different staining protocols (Table 1, scan ID 24–26). To allow contrast comparison without artifacts created by the scanning protocols, the intensities of each scan were standardized to Hounsfield Units (HU) thanks to distilled water phantoms scanned with the same parameters straight after each shrimp scan. Therefore, each sample was mounted as described above in plastic tubes, but this time above a liquid distilled water cell below as the standard. The water was scanned immediately after the sample without turning the X-ray beam off to ensure comparative signal stability. Hounsfield Units are a standard unit of X-ray CT density, in which air and water are ascribed values of 0 and 1,000, respectively. Calibration with water data points thus gives a useful and reproducible reference.

The HU values of each CNS organs and for the background were then collected as described above and the contrast ratio (*R*) was calculated with the means, as in the above Equation 1.

### 2.4 Segmentation of CNS organs

One optimal scan (scan ID 04) was manually segmented in Dragonfly to allow the visualization of each CNS organ in 3D and confirm the efficacy of the methodology for further comparative analyses. The volume of the following CNS organs were measured: lamina, external medulla, lobula, hemiellipsoid body neuropil, anterior medial protocerebral neuropil, posterior medial protocerebral neuropil, olfactory lobe, lateral antennular neuropil, antenna II neuropil.

## 3 Results

**Figure 2:**
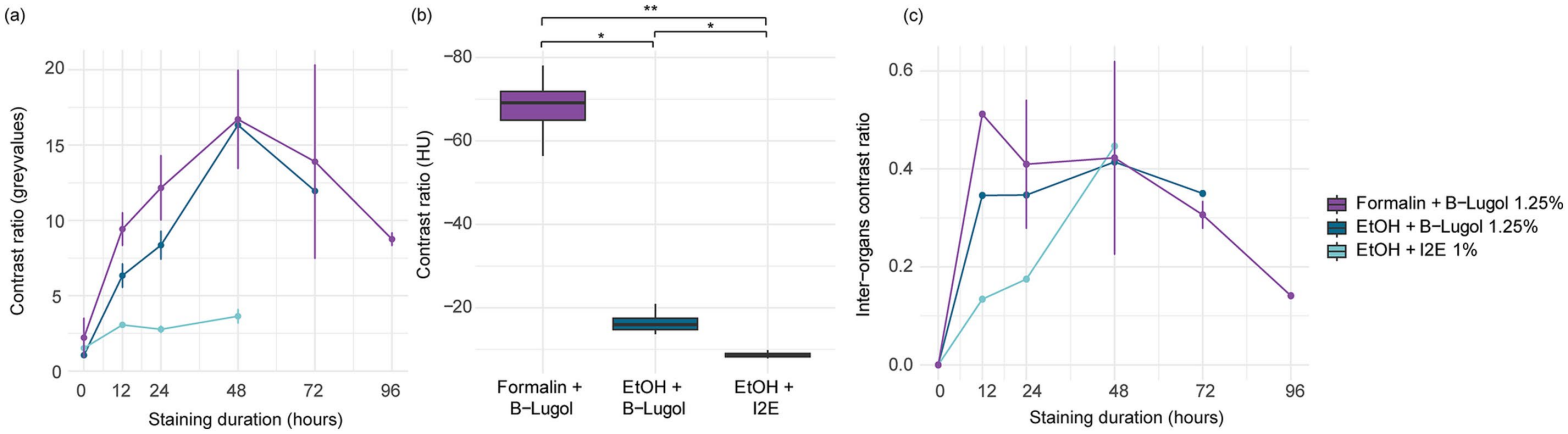
**(a)** Mean contrast ratio and **(c)** mean inter-organ contrast ratio, and their standard deviations (vertical bars) determined by measuring 16-bit gray values for *n* = 10 CNS organs for three fixation and staining protocols: formalin 10% and B-Lugol 1.25% (3 specimens), ethanol (EtOH) 95% and B-Lugol 1.25% (1 specimen), ethanol 95% and alcoholic iodine (I_2_E) 1% (1 specimen). **(b)** Boxplot of mean contrast ratios measured in Hounsfield Units on the CNS organs in standardized scans (ID 24–26) for three fixation and staining protocols: formalin 10% and B-Lugol 1.25% (1 specimen), ethanol (EtOH) 95% and B-Lugol 1.25% (1 specimen), ethanol 95% and alcoholic iodine (I_2_E) 1% (1 specimen). Significance is indicated with *p < 0.05 and **p < 0.01.

**Figure 3:**
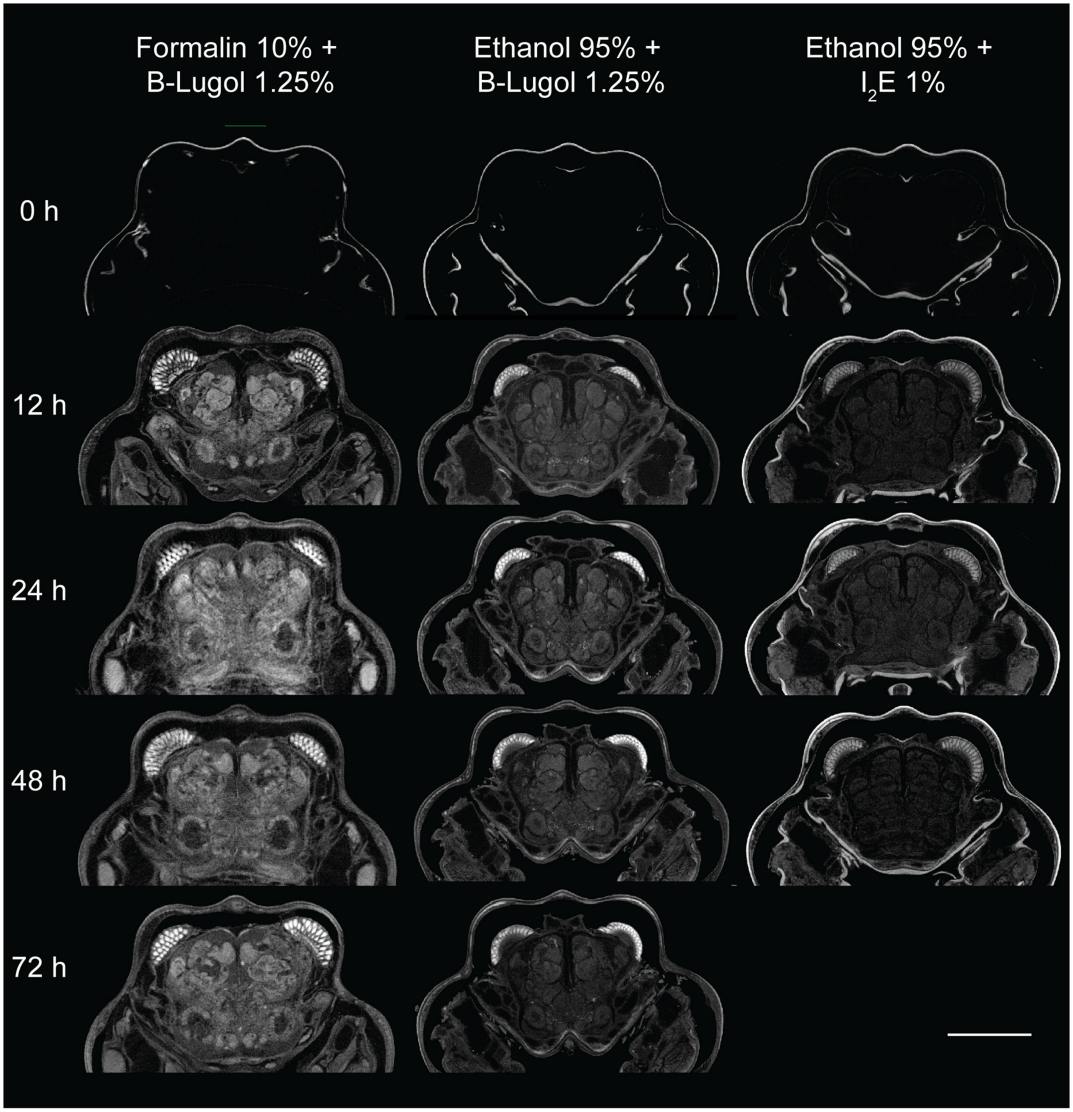
Examples of 2D slices of the brain region of the snapping shrimp *Alpheus richardsoni* across staining time points (0 – 72 h) for the three fixation and staining protocol used in this study. Scale bar = 1 mm.

The mean contrast ratio *R* of the CNS organs of *A. richardsoni* increased for each of the fixation and staining protocol until a peak staining time, before decreasing again (Figures 2a, 3 and Supplementary Table S1). Figure 3 shows example 2D slices sampled across time points for each protocol. Raw gray values (means and standard deviations) of all sampled CNS organs for all scans are presented in Supplementary Table S2. Peak staining time was 48 h for both B-Lugol 1.25% protocols, irrelevant of the fixation technique (formalin 10% or ethanol 95%). The contrast ratio *R* of both these protocols at peak staining time (48 h) showed no significant differences (*Z* = 0.30, *p adjusted* = 0.76). The ethanol + alcoholic iodine (I_2_E) protocol showed a very low contrast ratio overall, compared to the two others, and so was not pursued for a longer staining time. The inter-organ contrast ration *R_inter_* all showed a steep incline to 12 h, then leveling to peak at 48 h (Figure 2c and Supplementary Table S1). Similarly to *R*, *R_inter_* decreased after 48 h, indicating a reduced inter-organ distinguishability.

**Figure 4:**
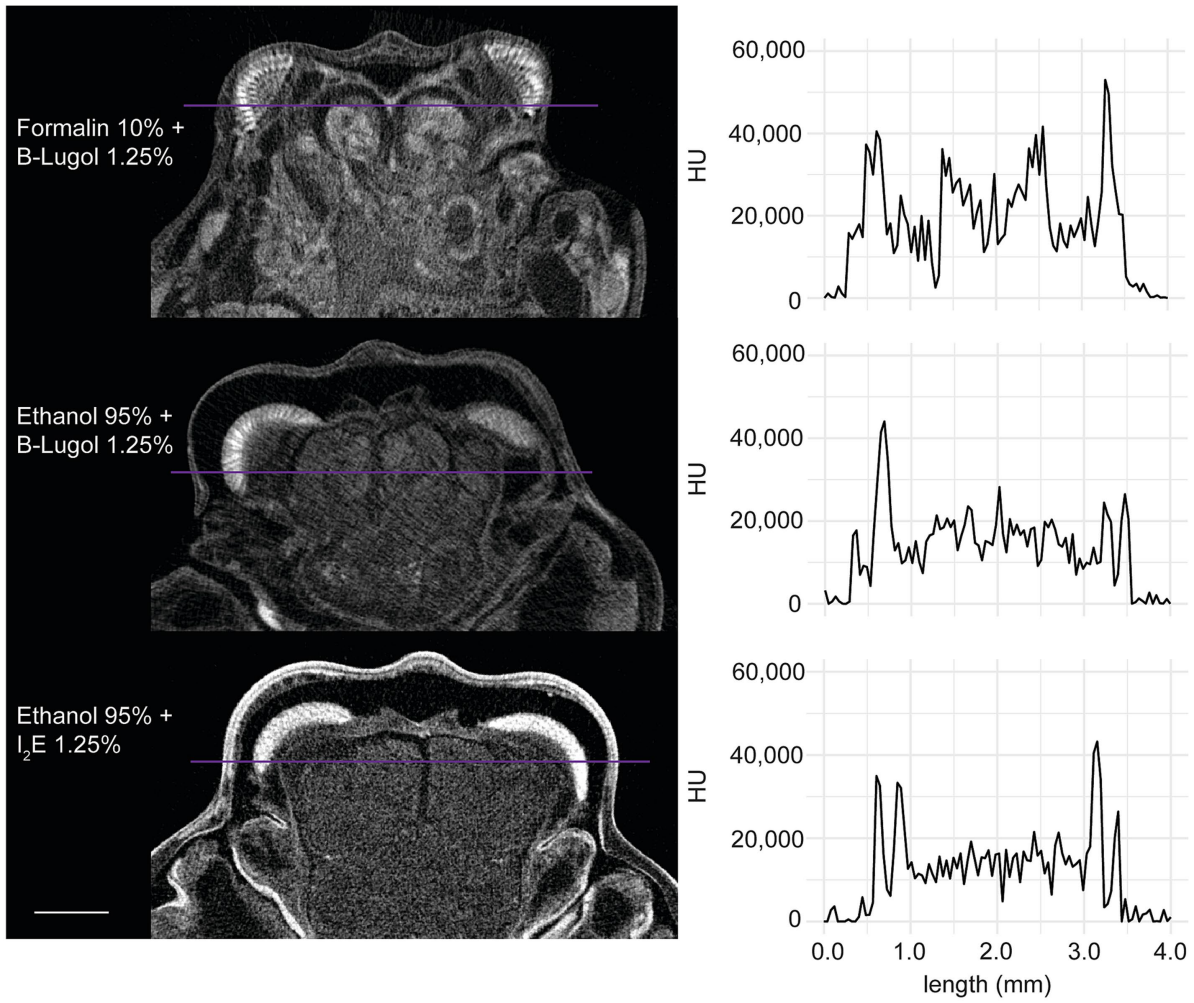
Example 2D slices (left) of the standardized scans for each combinations of fixation and staining protocol on the snapping shrimp *Alpheus richardsoni*. The plots (right) show the pixel intensity profile, in Hounsfield Units (HU), sampled across a line (in purple) of 4 mm in total. Scale bar = 1 mm.

In the standardized scans, contrast ratios for both B-Lugol protocols were significantly higher than the alcoholic iodine treatment (EtOH + B-Lugol: *Z* = −2.54, *p adjusted* < 0.05; Formalin + B-Lugol: *Z* = 5.08, *p adjusted* < 0.01; Figure 2b and Supplementary Table S3). For the B-Lugol contrast enhancement, the formalin fixation seems to clearly outperform the alcoholic fixation (*Z* = 2.54, *p adjusted* < 0.05; Figures 2b, 3). Figure 4 presents example 2D slices for the three scans, and an intensity line profile crossing the CNS: the formalin + B-Lugol typically showed a higher range of HU values, thus increasing the chance of delimitating brain organs (Figure 4 and Supplementary Table S4).

**Figure 5:**
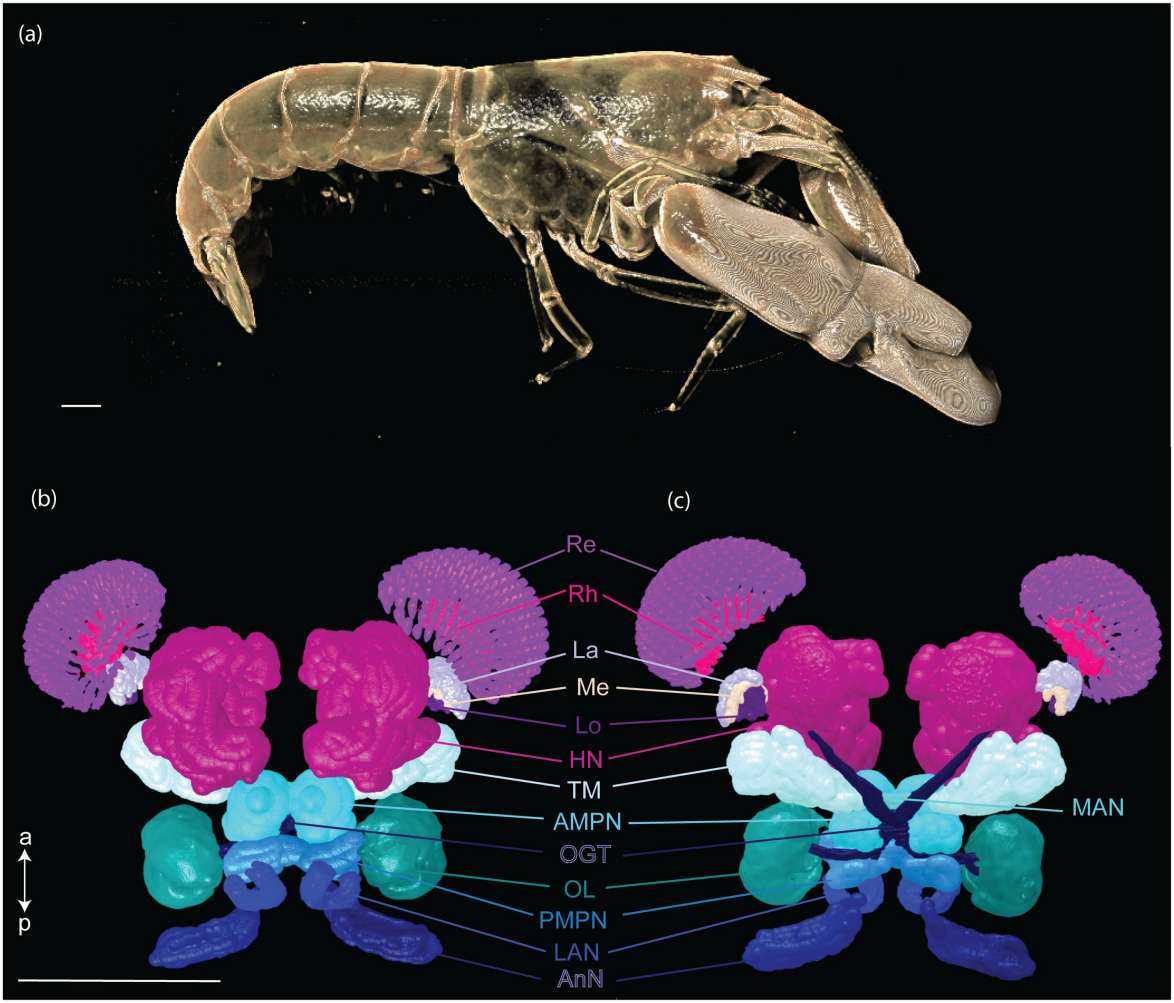
Three-dimensional reconstruction of the snapping shrimp, *Alpheus richardsoni*, (a) and its brain (b,c). Specimen (AR01, total length 23 mm) was fixed in 10% formalin and stained with B-Lugol 1.25% for 48 h, then μCT scanned (scan ID 04). Ventral view (b) and dorsal view (c). Scale bars = 1 mm. a: anterior, p: posterior. AMPN, anterior medial protocerebral neuropil; AnN, antenna II neuropil; HN, hemiellipsoid body neuropil; La, lamina; LAN, lateral antennular I neuropil; Lo, lobula; MAN, median antenullar neuropil; Me, external medulla; OGT, olfactory globular tract; OL, olfactory lobe; PMPN, posterior medial protocerebral neuropil; Re, retinula; Rh, rhabdom; TM, terminal medulla neuropil.

Fourteen CNS organs were successfully segmented (Figure 5 and Supplementary Video 1) from the optimal scan ID 4 (fixation in formalin 10% and stained with B-Lugol 1.25% for 48 h). Their measured surfaces and volumes are presented in Table 2.

**Table 2:** Three-dimensional measurements of segmented brain of a specimen (AR01) of the snapping shrimp *Alpheus richardsoni*, fixed in 10% formalin and stained with B-Lugol 1.25% for 48 h, then μCT scanned (scan ID 04).

Brain region	CNS organ	Surface (mm^2^)	Volume (mm^3^)	Percentage of total brain volume (%)	
Visual receptors	Retinula (Re)	9.0455	0.0709	11.54	14.33
	Rhabdom (Rh)	1.1306	0.0167	2.79	
Visual neuropils	Lamina (first visual neuropil) (La)	0.4719	0.0068	1.11	1.7
	External medulla (second visual neuropil) (Me)	0.2413	0.0036	0.59	
	Lobula (Lo)	0.1093	0.0014	0.23	
Protocerebrum	Hemiellipsoid body neuropil (HN)	4.2511	0.2924	47.61	66.14
	Terminal medulla neuropil (TM)	1.3099	0.0594	9.67	
	Anterior medial protocerebral neuropil (AMPN)	0.7948	0.0365	5.94	
	Posterior medial protocerebral neuropil (PMPN)	0.5265	0.0179	2.92	
Deutocerebrum	Olfactory lobe (OL)	2.0031	0.0590	9.61	11.75
	Lateral antennular I neuropil (LAN)	0.5809	0.0124	2.02	
	Median antenular neuropil (MAN)	0.0534	0.0007	0.12	
Tritocerebrum	Antenna II neuropil (AnN)	0.9321	0.0299	4.87	4.87
Tract	Olfactory globular tract (OGT)	0.5819	0.0066	1.07	1.07

Specimen total length was 23 mm.

## 4 Discussion

The present study demonstrates a novel application of a simple fixation and staining technique which facilitates X-ray tomography and the investigation of the nervous system of a model representative of a marine crustacean: the snapping shrimp, *Alpheus richardsoni*. The three methods presented here all enhanced contrast ratios (*R*) compared to unstained tissue. However, one combination of fixation and stain clearly provided a better contrast ratio than the others: the tissue was fixed with 10% formalin and subsequently stained with the water-based iodine B-Lugol 1.25%. B-Lugol, irrelevant of the fixation method used (i.e., 10% formalin or 95% ethanol), increased the contrast ratio through time until it peaked at 48 h, before reducing the contrast significantly, a sign of overstaining. Overstaining happens when all the tissues reach saturation (maximum absorption of iodine), therefore significantly reducing the contrast in between them. The inter-organ contrast ration (*R_inter_*) measuring the discriminability of CNS tissues with each other also increased through staining duration for all combinations, and after 48 h, were very similar to each other, before decreasing again due to overstaining. Alcoholic iodine (I_2_E), although still slightly enhancing the contrast, had a much poorer result compared to B-Lugol. Overall, the fixation with formalin and staining with B-Lugol offered optimal contrast in which to generate high quality micro-CT imagery for the investigation of crustacean CNS *in situ*.

Phosphate buffered 10% formalin is the most common fixative for crustaceans historically (Williams and Syoc, 2007; Martin, 2016). Our results demonstrate that it is particularly suited for the subsequent staining in water-based iodine. The caveat being, the solution becomes acidic and then reacts with CaCO_3_ components (i.e., typically the carapace of many crustaceans), making the specimen brittle and/or forms crystal artifacts (pers. observations). Therefore, fixation in formalin should be limited from a few hours to a few days for larger specimens (still long enough so that all internal tissues come in contact with the fixative). Following fixation, staining and scanning, the specimen should then be transferred in a preservation solution of 70% ethanol for long-term storage, by walking the ethanol concentration from 30 to 70% in 10–20% steps. While it was not observed in this study, air pockets can result from imperfect penetration of both fixative and staining agent, creating holes or voids in the micro-CT images, notably this typically occurs in much larger specimens than those scanned here (e.g., Gusmo et al., 2018). After scanning, the specimen can be ‘destained’ if needed by regularly replacing saturated preservative ethanol solution with a clear one, until the ethanol 70% remains completely clear (Gignac et al., 2016).

Formalin fixation may cross-link proteins and impede the stain intake, thus slowing down the staining of the tissue. For example, Swart et al. (2016) found that using 2% paraformaldehyde (i.e., similar to formalin, although formalin contains 4% formaldehyde) that stain uptake in *Calliphora* flies was approximately two times slower compared to ethanol fixation. In this study, we did not find that the formalin fixed shrimps were slower to uptake B-Lugol than the ethanol-fixed tissues (Figure 2). The size of the samples, the status of fixation (4% vs. 2%), the use of phosphate buffered saline to buffer the formalin, may have played a role.

While I_2_E has been reported as a successful staining agent for invertebrates fixed in alcohol (Metscher, 2009a; Nischik and Krieger, 2018; Machon et al., 2019; Wittfoth et al., 2019; Krieger and Spitzner, 2020) and for providing great contrast to vertebrate embryos (Metscher, 2009b), it proved much slower and less effective than water-based iodine for staining the shrimps in the present study. Therefore, for marine invertebrate specimen that are fixed and/or preserved in ethanol, we advise a step-wise downgrade to 30% ethanol before the use water-based iodine (B-Lugol) as a staining protocol for μCT scanning, rather than the use of I_2_E.

Iodine is known to cause soft-tissue shrinkage, notably due to the acidification of the solution, and the degree of shrinkage depends on the iodine concentration (Vickerton et al., 2013). To reduce this effect, we used buffered Lugol’s iodine (B-Lugol) to stabilize the pH of the staining solution and prevent heavy shrinkage (Dawood et al., 2021). While we did not investigate whether the soft tissues of interest (i.e., CNS) in this study were affected, some remaining shrinkage may be expected. Another popular contrast agent is phosphotungstic acid (PTA), which, prepared in an aqueous solution, seems to provide less tissue shrinkage compared to iodine (Buytaert et al., 2014). PTA has also been used to successfully stain soft tissues in invertebrates, including from the CNS (Faulwetter et al., 2013a; Smith et al., 2016; Swart et al., 2016; Sakurai and Ikeda, 2019; Rivera-Quiroz and Miller, 2022; Ikenaga et al., 2024). However, one of the reasons why PTA was not trialed in this study, and may be generally less used than iodine, is the apparent slower tissue penetration (Buytaert et al., 2014; Rivera-Quiroz and Miller, 2022). However, PTA may also offer increased discriminability between tissues of different types (Swart et al., 2016). Further studies are required to compare the efficacy of PTA and the relative staining time needed for optimal contrast ratio and for the discrimination of CNS tissues from marine crustaceans. Similarly, more work needs to determine the shrinkage induced between buffered iodine solutions (like B-Lugol) and PTA, at different concentrations.

The optimal fixation and staining methodology in the current study allowed the successful semi-automatic segmentation of the CNS organs in *A. richardsoni*, and volumetric measurements. One of the overarching questions of evolutionary neuroscience is how neuroanatomy reflects the sensory output that the brain processes and hence mirrors the sensory landscape that animals analyze (Sandeman et al., 1993, 2014; Strausfeld, 2012; Meth et al., 2017). Such comparative and functional studies require calibrated data of the anatomical structures in focus (e.g., neuropils in the brain of invertebrates) within an anatomical context as close to the natural state as possible, which a non-invasive technique like X-ray μCT can offer (Sombke et al., 2015). While available studies on marine crustaceans have mostly used traditional neuroanatomical methods like histology, immunohistochemistry and confocal laser-scan microscopy (Krieger et al., 2012; Polanska et al., 2012; e.g., Kenning and Harzsch, 2013), some recent work has included μCT data like the present study (Meth et al., 2017; Machon et al., 2019; Krieger and Spitzner, 2020; Krieger et al., 2020). Collated together, these detailed descriptions and measurements of the neuroanatomy of different species represent a significant step toward linking morphology to function. Our methodology entails fewer and less time-consuming steps than more traditional methods, and therefore could be replicated on larger sample sizes, even at a population level. It thus opens the possibility of looking at the effects of external factors like anthropogenic threats which may impact the anatomical integrity of the central and peripheral nervous system of these organisms (Kelley et al., 2018).

The brain of *A. richardsoni* can still be subdivided into an anterolateral portion, the visual receptors and the visual neuropils, and a medioventral portion within the cephalothorax, with the protocerebrum (66% of segmented volume), deutocerebrum (12%) and tritocerebrum (5%) (Figure 5 and Table 2). Snapping shrimps are part of the Alpheidae family, known for their wide range of social associations and behaviours (Chak et al., 2015), and notably as the only marine invertebrate taxon that have developed eusociality (Duffy, 1996). Alpheids are also the most ubiquitous sound-producing animals in the marine environment, responsible for the typical crackling sounds heard in temperate and tropical reefs around the world (Radford et al., 2008; Bohnenstiehl et al., 2016; Lillis and Mooney, 2018). *Alpheus richardsoni* is known to detect the particle motion of the sound (Dinh and Radford, 2021) and *A. heterochaelis* to have the fastest sampling eyes ever described in an aquatic animal (Kingston et al., 2020). Therefore, we expect high sensory processing abilities to receive and output social and communicative signals. The large protocerebrum present in *A. richardsoni* includes the higher order neuropils (hemiellipsoid body and terminal medulla) receiving multimodal inputs from primary processing units, such as the visual and olfactory neuropils. These higher order brain centers are thought to be integrative and providing the neuronal substrate for sophisticated behaviors involving 3D spatial perception (visual, mechanical or olfactory) (Sandeman et al., 2014; Krieger et al., 2020).

The development of tissue preparation and μCT scanning protocols emerge as a promising new strategy for investigating comparative neuroanatomical questions in all animal taxa (Jonsson, 2023; Collin et al., 2024). While it cannot not replace the fine images produced by histology, immunohistochemistry and confocal laser scanning microscopy, μCT enables non-destructive imaging of the CNS of small organisms *in situ* as a method of choice to develop detailed neuroanatomical atlases in key model species (e.g., the bumblebee, Smith et al., 2016; Rother et al., 2021) and enhancing comparative analyses between species (Sombke et al., 2015). MicroCT can also help elucidate the factors elicited by anthropogenic and environmental changes affecting physiology and behavior. It is a quantitative and rapid way to measure the volumes of organs and visualize anatomical abnormalities (Brinkmann et al., 2016; Wlodkowic et al., 2022). It can facilitate new studies on anatomical variations not only in overall brain size, but also different parts of the nervous system upon exposure to pollutants and neurotoxins. Although this application is still relatively untapped in invertebrate studies, microCT was recently used to show the negative effect of arsenic exposure on the development of the olfactory neuropils in honey bees (Monchanin et al., 2024). The digital nature of 3D data also allow for the application of automated shape analyses (e.g., geometric morphometrics) to interrogate the structure-to-function relationships, where variations in neural structures can be mathematically modeled (e.g., finite element analysis) (Chapuis et al., 2023).

To conclude, our iodine-based approach provided a cost-effective and time-efficient toolkit to enable a wide range of studies exploring intra- and inter-specific variability of soft tissues (in this case the CNS) of marine crustaceans. It reduces negative impacts on specimen preservation by eliminating the use of a drying step, which can be especially useful for museum collections. Given the important ecosystem services provided by marine crustaceans and their notable declines due to habitat loss and climate change (Behringer and Duermit-Moreau, 2021), our methodology could help elucidate the factors and stressors affecting the development of the nervous system or other soft tissues (e.g., muscles, peripheral sensory organs, digestive and respiratory systems), and their functional anatomy.

## Data Availability

The original contributions presented in the study are included in the article/Supplementary material, further inquiries can be directed to the corresponding author.
