# Supplementary material for: Bioimaging marine crustacean brain: quantitative comparison of micro-CT preparations in an Alpheid snapping shrimp
Source: Front Neurosci. 2024 Nov 26;18:1428825. doi: 10.3389/fnins.2024.1428825 (PMC11628493; doi:10.3389/fnins.2024.1428825)
Supplement: Supplementary file 1 [file Table_1.docx]

***Supplementary Material***

Table S1: Mean contrast ratio *R* (averaged from all CNS organs), inter-organ contrast ratio *R_inter_*_,_ and standard deviations (SD) for each fixation and staining agent combinations.

| Scan(s) ID | Specimen(s) ID | Fixation | Staining agent | Staining time (hours) | Contrast ratio mean *R* | *R* SD | Inter-organ contrast ratio *R_inter_* | *R_inter SD_* |
| --- | --- | --- | --- | --- | --- | --- | --- | --- |
| 01, 06, 10 | AR01, AR02, AR03 | Formalin 10% | B-Lugol 1.25% | 0 | 2.219 | 1.301 | 0.000 | 0.000 |
| 02 | AR01 | Formalin 10% | B-Lugol 1.25% | 12 | 9.425 | 1.092 | 0.512 | NA |
| 03, 07, 11 | AR01, AR02, AR03 | Formalin 10% | B-Lugol 1.25% | 24 | 12.161 | 2.144 | 0.410 | 0.131 |
| 04, 08, 12 | AR01, AR02, AR03 | Formalin 10% | B-Lugol 1.25% | 48 | 16.709 | 3.262 | 0.423 | 0.197 |
| 05, 09, 13 | AR01, AR02, AR03 | Formalin 10% | B-Lugol 1.25% | 72 | 13.901 | 6.432 | 0.306 | 0.027 |
| 14 | AR03 | Formalin 10% | B-Lugol 1.25% | 96 | 8.757 | 0.420 | 0.141 | NA |
| 15 | AR04 | EtOH 95% | I_2_E 1% | 0 | 1.523 | 0.000 | 0.000 | NA |
| 16 | AR04 | EtOH 95% | I_2_E 1% | 12 | 3.066 | 0.168 | 0.134 | NA |
| 17 | AR04 | EtOH 95% | I_2_E 1% | 24 | 2.772 | 0.226 | 0.175 | NA |
| 18 | AR04 | EtOH 95% | I_2_E 1% | 48 | 3.641 | 0.452 | 0.447 | NA |
| 19 | AR05 | EtOH 95% | B-Lugol 1.25% | 0 | 1.055 | 0.000 | 0.000 | NA |
| 20 | AR05 | EtOH 95% | B-Lugol 1.25% | 12 | 6.340 | 0.789 | 0.346 | NA |
| 21 | AR05 | EtOH 95% | B-Lugol 1.25% | 24 | 8.356 | 0.936 | 0.347 | NA |
| 22 | AR05 | EtOH 95% | B-Lugol 1.25% | 48 | 16.330 | 1.878 | 0.414 | NA |
| 23 | AR05 | EtOH 95% | B-Lugol 1.25% | 72 | 11.953 | 1.3407 | 0.350 | NA |

Table S2: Mean and standard deviation (SD) greyvalues (16-bit) from the histogram of each CNS organ sampled in each scan. Where the contrast was high enough, areas from both left and right organs were sampled and then averaged. Two background values were taken from two surface areas for each scan. AMPN: anterior medial protocerebral neuropil, AnN: antenna II neuropil, HN: hemiellipsoid body neuropil, La: lamina, LAN: lateral antennular I neuropil, Lo: lobula, Me: external medulla, OL: olfactory lobe, PMPN posterior medial protocerebral neuropil, TM: terminal medulla neuropil.

| **Scan ID** | **Side** | **La** | | **Me** | | **Lo** | | **HN** | | **TM** | | **AMPN** | | **PMPN** | | **OL** | | **LAN** | | **AnN** | | **Background** | |
| --- | --- | --- | --- | --- | --- | --- | --- | --- | --- | --- | --- | --- | --- | --- | --- | --- | --- | --- | --- | --- | --- | --- | --- |
|  |  | **Mean** | **SD** | **Mean** | **SD** | **Mean** | **SD** | **Mean** | **SD** | **Mean** | **SD** | **Mean** | **SD** | **Mean** | **SD** | **Mean** | **SD** | **Mean** | **SD** | **Mean** | **SD** | **Mean** | **SD** |
| 01 | L |  |  |  |  |  |  |  |  |  |  |  |  |  |  |  |  |  |  |  |  | 40 | 105 |
|  | R |  |  |  |  |  |  |  |  |  |  |  |  |  |  |  |  |  |  |  |  | 3143 | 1190 |
|  | Mean | 5133 | 1057 | 5133 | 1057 | 5133 | 1057 | 5133 | 1057 | 5133 | 1057 | 5133 | 1057 | 5133 | 1057 | 5133 | 1057 | 5133 | 1057 | 5133 | 1057 | 1592 | 647 |
| 02 | L | 37549 | 3223 | 34850 | 2244 | 35205 | 2617 | 33680 | 2220 | 32629 | 2571 | 31647 | 2633 | 25711 | 2453 | 32547 | 3040 | 35082 | 2401 | 34389 | 3011 | 2587 | 2214 |
|  | R | 42953 | 2878 | 35681 | 3018 | 35682 | 3204 | 34355 | 2686 | 34138 | 2836 | 31535 | 2190 | 27535 | 2645 | 29424 | 3353 | 33895 | 2348 | 34700 | 2649 | 3871 | 1505 |
|  | Mean | 40251 | 3051 | 35265 | 2631 | 35443 | 2911 | 34018 | 2453 | 33384 | 2704 | 31591 | 2412 | 26623 | 2549 | 30986 | 3196 | 34488 | 2374 | 34545 | 2830 | 3229 | 1860 |
| 03 | L | 21343 | 1546 | 20171 | 1950 | 19042 | 1778 | 19677 | 1391 | 19661 | 1549 | 17349 | 1268 | 13573 | 1551 | 16964 | 1265 | 19432 | 1329 | 19198 | 1559 | 1896 | 1012 |
|  | R | 21940 | 1439 | 19901 | 1471 | 20124 | 1299 | 18251 | 1249 | 18208 | 1151 | 16403 | 1432 | 14529 | 1616 | 17312 | 1586 | 18808 | 1459 | 17940 | 1798 | 1001 | 1013 |
|  | Mean | 21641 | 1492 | 20036 | 1711 | 19583 | 1538 | 18964 | 1320 | 18935 | 1350 | 16876 | 1350 | 14051 | 1583 | 17138 | 1426 | 19120 | 1394 | 18569 | 1678 | 1449 | 1012 |
| 04 | L | 37140 | 2937 | 33909 | 2587 | 30228 | 2287 | 30668 | 2236 | 30439 | 3398 | 29924 | 2490 | 21129 | 2467 | 28900 | 3001 | 28695 | 2517 | 28438 | 2501 | 1442 | 1599 |
|  | R | 35892 | 2622 | 31719 | 2566 | 34272 | 2857 | 29365 | 2719 | 26546 | 2898 | 28935 | 2454 | 23215 | 2159 | 28422 | 2287 | 31173 | 2178 | 19123 | 2920 | 1331 | 1548 |
|  | Mean | 36516 | 2780 | 32814 | 2576 | 32250 | 2572 | 30016 | 2477 | 28492 | 3148 | 29430 | 2472 | 22172 | 2313 | 28661 | 2644 | 29934 | 2348 | 23781 | 2711 | 1387 | 1573 |
| 05 | L | 24545 | 2101 | 22931 | 2043 | 22520 | 2353 | 27104 | 2151 | 26221 | 2417 | 22368 | 2013 | 19821 | 1941 | 23088 | 2077 | 24910 | 1977 | 24882 | 2251 | 467 | 830 |
|  | R | 22027 | 1935 | 21124 | 1567 | 23224 | 2160 | 25155 | 1878 | 23680 | 2035 | 22666 | 1971 | 20434 | 2329 | 22490 | 2042 | 25181 | 2385 | 24373 | 2026 | 1555 | 1292 |
|  | Mean | 23286 | 2018 | 22027 | 1805 | 22872 | 2257 | 26129 | 2015 | 24951 | 2226 | 22517 | 1992 | 20127 | 2135 | 22789 | 2060 | 25046 | 2181 | 24628 | 2139 | 1011 | 1061 |
| 06 | L |  |  |  |  |  |  |  |  |  |  |  |  |  |  |  |  |  |  |  |  | 166 | 315 |
|  | R |  |  |  |  |  |  |  |  |  |  |  |  |  |  |  |  |  |  |  |  | 1798 | 1215 |
|  | Mean | 4699 | 1313 | 4699 | 1313 | 4699 | 1313 | 4699 | 1313 | 4699 | 1313 | 4699 | 1313 | 4699 | 1313 | 4699 | 1313 | 4699 | 1313 | 4699 | 1313 | 982 | 765 |
| 07 | L | 34249 | 3426 | 30653 | 3358 | 30554 | 3300 | 34788 | 2824 | 27531 | 2923 | 29275 | 2706 | 25452 | 2986 | 24738 | 2634 | 32630 | 2751 | 30698 | 3112 | 2764 | 1810 |
|  | R | 30800 | 3721 | 32115 | 3368 | 30120 | 2724 | 35033 | 3402 | 30044 | 2988 | 27708 | 2943 | 25667 | 2841 | 24781 | 3620 | 35197 | 3066 | 28919 | 2871 | 2571 | 2179 |
|  | Mean | 32524 | 3574 | 31384 | 3363 | 30337 | 3012 | 34911 | 3113 | 28787 | 2955 | 28491 | 2824 | 25560 | 2913 | 24760 | 3127 | 33914 | 2908 | 29808 | 2992 | 2667 | 1994 |
| 08 | L | 39601 | 3896 | 39145 | 3122 | 39354 | 3220 | 40523 | 3131 | 36756 | 3305 | 34553 | 3209 | 32091 | 3301 | 36706 | 3580 | 40584 | 3067 | 37480 | 3355 | 2584 | 2216 |
|  | R | 39471 | 3662 | 37753 | 3660 | 37340 | 3506 | 38651 | 3182 | 36983 | 3119 | 37866 | 3595 | 30117 | 3385 | 36141 | 2643 | 38956 | 3363 | 39122 | 3477 | 2372 | 2136 |
|  | Mean | 39536 | 3779 | 38449 | 3391 | 38347 | 3363 | 39587 | 3157 | 36869 | 3212 | 36209 | 3402 | 31104 | 3343 | 36423 | 3111 | 39770 | 3215 | 38301 | 3416 | 2478 | 2176 |
| 09 | L | 35401 | 2876 | 32247 | 2269 | 32446 | 1976 | 32747 | 2179 | 32193 | 2311 | 29619 | 2675 | 27009 | 3716 | 29634 | 2557 | 32418 | 2220 | 30564 | 2378 | 2504 | 1764 |
|  | R | 33424 | 2501 | 33103 | 2131 | 31025 | 2949 | 33218 | 2141 | 25639 | 2205 | 29654 | 2347 | 24479 | 2134 | 30602 | 3203 | 30677 | 2739 | 30544 | 2303 | 2130 | 1410 |
|  | Mean | 34412 | 2689 | 32675 | 2200 | 31735 | 2462 | 32983 | 2160 | 28916 | 2258 | 29636 | 2511 | 25744 | 2925 | 30118 | 2880 | 31548 | 2479 | 30554 | 2340 | 2317 | 1587 |
| 10 | L | 27179 | 2062 | 26513 | 1977 | 25118 | 1569 | 24708 | 1695 | 23048 | 2247 | 22560 | 2477 | 24322 | 2562 | 23773 | 1943 | 24207 | 1982 | 23841 | 1772 | 2698 | 1709 |
|  | R | 25542 | 2560 | 24289 | 2025 | 23845 | 1956 | 24409 | 2010 | 23349 | 1759 | 23636 | 2237 | 22604 | 2238 | 23418 | 2839 | 23816 | 2141 | 23505 | 2627 | 2259 | 1930 |
|  | Mean | 26360 | 2311 | 25401 | 2001 | 24482 | 1762 | 24558 | 1853 | 23198 | 2003 | 23098 | 2357 | 23463 | 2400 | 23596 | 2391 | 24012 | 2061 | 23673 | 2199 | 2479 | 1819 |
| 11 | L |  |  |  |  |  |  |  |  |  |  |  |  |  |  |  |  |  |  |  |  | 6126 | 4953 |
|  | R |  |  |  |  |  |  |  |  |  |  |  |  |  |  |  |  |  |  |  |  | 1887 | 917 |
|  | Mean | 6611 | 2012 | 6611 | 2012 | 6611 | 2012 | 6611 | 2012 | 6611 | 2012 | 6611 | 2012 | 6611 | 2012 | 6611 | 2012 | 6611 | 2012 | 6611 | 2012 | 4007 | 2935 |
| 12 | L | 37027 | 2603 | 33402 | 3426 | 31280 | 2781 | 34738 | 2409 | 29890 | 2248 | 31344 | 3115 | 29225 | 3004 | 33429 | 2252 | 33065 | 2854 | 34540 | 2362 | 1652 | 1444 |
|  | R | 35647 | 3489 | 35225 | 2624 | 31925 | 2076 | 34013 | 2229 | 30314 | 2855 | 32965 | 2358 | 27602 | 2468 | 31562 | 2697 | 33300 | 2386 | 34533 | 2682 | 2586 | 2031 |
|  | Mean | 36337 | 3046 | 34314 | 3025 | 31602 | 2428 | 34376 | 2319 | 30102 | 2552 | 32154 | 2737 | 28414 | 2736 | 32495 | 2475 | 33182 | 2620 | 34537 | 2522 | 2119 | 1737 |
| 13 | L | 43729 | 3379 | 36945 | 2180 | 35812 | 2125 | 39000 | 2050 | 32166 | 2218 | 35833 | 2100 | 32462 | 2853 | 36888 | 3015 | 35111 | 2447 | 35539 | 2585 | 2072 | 1743 |
|  | R | 41145 | 2597 | 40181 | 2770 | 36114 | 1932 | 39298 | 2237 | 31056 | 2180 | 37346 | 2876 | 32138 | 2636 | 32910 | 2043 | 35854 | 3598 | 36199 | 2923 | 2246 | 1645 |
|  | Mean | 42437 | 2988 | 38563 | 2475 | 35963 | 2029 | 39149 | 2143 | 31611 | 2199 | 36590 | 2488 | 32300 | 2744 | 34899 | 2529 | 35482 | 3023 | 35869 | 2754 | 2159 | 1694 |
| 14 | L | 34628 | 1839 | 30943 | 1702 | 30474 | 2203 | 29388 | 1369 | 28057 | 1937 | 29323 | 1644 | 30589 | 1773 | 29025 | 1685 | 31108 | 1974 | 30119 | 1817 | 4764 | 1711 |
|  | R | 37468 | 1642 | 30737 | 1725 | 30349 | 2169 | 30230 | 2097 | 28096 | 1711 | 29676 | 1767 | 30976 | 1965 | 29464 | 1684 | 28849 | 1808 | 29850 | 2294 | 2656 | 1759 |
|  | Mean | 36048 | 1741 | 30840 | 1713 | 30412 | 2186 | 29809 | 1733 | 28077 | 1824 | 29499 | 1705 | 30782 | 1869 | 29245 | 1685 | 29979 | 1891 | 29984 | 2055 | 3710 | 1735 |
| 15 | L |  |  |  |  |  |  |  |  |  |  |  |  |  |  |  |  |  |  |  |  | 461 | 490 |
|  | R |  |  |  |  |  |  |  |  |  |  |  |  |  |  |  |  |  |  |  |  | 1996 | 556 |
|  | Mean | 3099 | 638 | 3099 | 638 | 3099 | 638 | 3099 | 638 | 3099 | 638 | 3099 | 638 | 3099 | 638 | 3099 | 638 | 3099 | 638 | 3099 | 638 | 1228 | 523 |
| 16 | L | 7885 | 820 | 8132 | 645 | 7442 | 1031 | 7265 | 625 | 8084 | 858 | 7103 | 885 | 7691 | 875 | 6505 | 784 | 7966 | 1001 | 8358 | 981 | 1452 | 829 |
|  | R | 8161 | 989 | 7686 | 906 | 7342 | 820 | 7516 | 660 | 7195 | 858 | 7762 | 909 | 7009 | 1025 | 7643 | 806 | 7940 | 985 | 7222 | 1024 | 2284 | 891 |
|  | Mean | 8023 | 904 | 7909 | 776 | 7392 | 925 | 7390 | 643 | 7640 | 858 | 7433 | 897 | 7350 | 950 | 7074 | 795 | 7953 | 993 | 7790 | 1002 | 1868 | 860 |
| 17 | L | 8623 | 726 | 8033 | 787 | 7636 | 802 | 7933 | 840 | 8007 | 980 | 7335 | 598 | 7178 | 996 | 7165 | 647 | 7469 | 704 | 7925 | 720 | 1825 | 1035 |
|  | R | 8279 | 665 | 8164 | 766 | 7477 | 961 | 8181 | 645 | 8223 | 867 | 7310 | 807 | 7465 | 1019 | 7219 | 907 | 7991 | 676 | 8965 | 1008 | 2326 | 808 |
|  | Mean | 8451 | 695 | 8099 | 776 | 7557 | 882 | 8057 | 742 | 8115 | 923 | 7323 | 702 | 7321 | 1008 | 7192 | 777 | 7730 | 690 | 8445 | 864 | 2075 | 922 |
| 18 | L | 11929 | 961 | 10816 | 1001 | 11220 | 1234 | 12670 | 1476 | 11655 | 1070 | 10870 | 1100 | 7784 | 1466 | 10740 | 1121 | 10902 | 981 | 12093 | 1431 | 1681 | 1397 |
|  | R | 12428 | 1170 | 11421 | 1023 | 11092 | 977 | 12344 | 1166 | 10980 | 1236 | 10653 | 1001 | 9507 | 1287 | 11140 | 1345 | 11139 | 1288 | 12515 | 1487 | 3144 | 1284 |
|  | Mean | 12179 | 1066 | 11119 | 1012 | 11156 | 1105 | 12507 | 1321 | 11317 | 1153 | 10762 | 1050 | 8646 | 1376 | 10940 | 1233 | 11020 | 1135 | 12304 | 1459 | 2412 | 1340 |
| 19 | L |  |  |  |  |  |  |  |  |  |  |  |  |  |  |  |  |  |  |  |  | 642 | 618 |
|  | R |  |  |  |  |  |  |  |  |  |  |  |  |  |  |  |  |  |  |  |  | 3297 | 817 |
|  | Mean | 4048 | 886 | 4048 | 886 | 4048 | 886 | 4048 | 886 | 4048 | 886 | 4048 | 886 | 4048 | 886 | 4048 | 886 | 4048 | 886 | 4048 | 886 | 1970 | 718 |
| 20 | L | 23482 | 1757 | 21010 | 1916 | 18887 | 1827 | 22618 | 2297 | 18700 | 1942 | 19002 | 2050 | 25292 | 2599 | 20681 | 2462 | 20334 | 1996 | 18045 | 1618 | 1989 | 1299 |
|  | R | 22389 | 2244 | 19937 | 1882 | 20004 | 1557 | 24386 | 1876 | 19806 | 1855 | 18918 | 1633 | 25746 | 3103 | 21211 | 1995 | 18878 | 1798 | 20199 | 2594 | 3727 | 1567 |
|  | Mean | 22935 | 2001 | 20473 | 1899 | 19446 | 1692 | 23502 | 2086 | 19253 | 1899 | 18960 | 1842 | 25519 | 2851 | 20946 | 2229 | 19606 | 1897 | 19122 | 2106 | 2858 | 1433 |
| 21 | L | 21658 | 2186 | 20988 | 1861 | 18487 | 1752 | 19809 | 1556 | 18581 | 1862 | 17634 | 2247 | 23962 | 2736 | 20073 | 2054 | 18594 | 1286 | 18058 | 1831 | 2170 | 1511 |
|  | R | 23480 | 1690 | 20679 | 2278 | 17490 | 1816 | 20413 | 1724 | 18207 | 1695 | 19080 | 1801 | 24491 | 4123 | 20102 | 2007 | 19863 | 1483 | 19699 | 1816 | 2120 | 1278 |
|  | Mean | 22569 | 1938 | 20833 | 2069 | 17989 | 1784 | 20111 | 1640 | 18394 | 1778 | 18357 | 2024 | 24226 | 3430 | 20088 | 2030 | 19228 | 1385 | 18878 | 1824 | 2145 | 1395 |
| 22 | L | 17651 | 1354 | 17133 | 1731 | 14406 | 1237 | 16381 | 1308 | 15577 | 1439 | 13652 | 1833 | 19904 | 2510 | 15368 | 1745 | 14122 | 1726 | 13749 | 1349 | 1034 | 961 |
|  | R | 15725 | 1428 | 15974 | 1358 | 15484 | 1413 | 17482 | 1591 | 15858 | 1240 | 14470 | 1789 | 19868 | 2912 | 14759 | 1412 | 14022 | 1390 | 16750 | 1773 | 803 | 943 |
|  | Mean | 16688 | 1391 | 16554 | 1545 | 14945 | 1325 | 16931 | 1449 | 15717 | 1339 | 14061 | 1811 | 19886 | 2711 | 15063 | 1579 | 14072 | 1558 | 15250 | 1561 | 918 | 952 |
| 23 | L | 16444 | 1172 | 14480 | 1224 | 14337 | 1442 | 15722 | 1312 | 14224 | 1557 | 13841 | 1322 | 18901 | 1805 | 15506 | 1382 | 13713 | 1375 | 13100 | 1510 | 1112 | 1080 |
|  | R | 17381 | 1484 | 15753 | 1688 | 14036 | 1250 | 17395 | 1295 | 14821 | 1525 | 12918 | 1668 | 17223 | 3421 | 14992 | 1616 | 14009 | 1441 | 14093 | 1520 | 1226 | 1059 |
|  | Mean | 16912 | 1328 | 15116 | 1456 | 14186 | 1346 | 16558 | 1304 | 14522 | 1541 | 13380 | 1495 | 18062 | 2613 | 15249 | 1499 | 13861 | 1408 | 13596 | 1515 | 1169 | 1069 |

Table S3: Mean contrast ratios measured from Hounsfield Units in scans 24 – 26 for each fixation and staining agent combinations.

.

| Scan(s) ID | Specimen(s) ID | Fixation | Staining agent | Staining time (hours) | Mean contrast ratio | Contrast ratio SD |
| --- | --- | --- | --- | --- | --- | --- |
| 24 | AR06 | Formalin 10% | B-Lugol 1.25% | 48 | -68.641 | 6.829 |
| 25 | AR07 | EtOH 95% | I_2_EtOH | 48 | -8.740 | 0.591 |
| 26 | AR08 | EtOH 95% | B-Lugol 1.25% | 48 | -16.305 | 2.257 |

Table S4: Mean and standard deviation (SD) Hounsfield Unites (HU) from the histogram of each CNS organ sampled in scan 24 - 25. AMPN: anterior medial protocerebral neuropil, AnN: antenna II neuropil, HN: hemiellipsoid body neuropil, La: lamina, LAN: lateral antennular I neuropil, Lo: lobula, Me: external medulla, OL: olfactory lobe, PMPN posterior medial protocerebral neuropil, TM: terminal medulla neuropil.

| **Scan ID** | **Side** | **La** | | **Me** | | **Lo** | | **HN** | | **TM** | | **AMPN** | | **PMPN** | | **OL** | | **LAN** | | **AnN** | | **Background** | |
| --- | --- | --- | --- | --- | --- | --- | --- | --- | --- | --- | --- | --- | --- | --- | --- | --- | --- | --- | --- | --- | --- | --- | --- |
|  |  | **Mean** | **SD** | **Mean** | **SD** | **Mean** | **SD** | **Mean** | **SD** | **Mean** | **SD** | **Mean** | **SD** | **Mean** | **SD** | **Mean** | **SD** | **Mean** | **SD** | **Mean** | **SD** | **Mean** | **SD** |
| 24 | L | 13031 | 1760 | 11341 | 1459 | 10623 | 1526 | 12748 | 1380 | 11194 | 1172 | 12429 | 1600 | 9341 | 1528 | 11065 | 1429 | 9496 | 1778 | 12169 | 1410 | -315 | 807 |
|  | R | 12979 | 1799 | 12567 | 2009 | 10753 | 1858 | 13094 | 1543 | 11042 | 1473 | 11487 | 1537 | 9364 | 1575 | 11670 | 1472 | 10766 | 1770 | 11106 | 1756 | -23 | 1024 |
|  | Mean | 13005 | 1779 | 11954 | 1734 | 10688 | 1692 | 12921 | 1462 | 11118 | 1323 | 11958 | 1569 | 9352 | 1552 | 11367 | 1450 | 10131 | 1774 | 11637 | 1583 | -169 | 915 |
| 25 | L | 3439 | 624 | 2849 | 1180 | 6737 | 455 | 3070 | 674 | 3132 | 561 | 3014 | 780 | 2815 | 812 | 3041 | 561 | 3014 | 572 | 3271 | 629 | -514 | 405 |
|  | R | 3663 | 552 | 3254 | 579 | 3398 | 681 | 3630 | 491 | 3174 | 379 | 2502 | 646 | 3055 | 610 | 2803 | 689 | 2817 | 702 | 3118 | 811 | -289 | 459 |
|  | Mean | 3551 | 588 | 3052 | 637 | 3062 | 568 | 3350 | 583 | 3153 | 470 | 2758 | 713 | 2935 | 711 | 2922 | 625 | 2915 | 637 | 3195 | 720 | -401 | 432 |
| 26 | L | 7735 | 1209 | 6806 | 1734 | 10688 | 1535 | 7804 | 1074 | 6103 | 1063 | 5913 | 1480 | 8221 | 1076 | 7228 | 1065 | 5680 | 1215 | 6497 | 1367 | -589 | 508 |
|  | R | 7739 | 1305 | 7162 | 1169 | 6855 | 1186 | 8137 | 1378 | 6253 | 1019 | 5743 | 904 | 10040 | 1123 | 6664 | 1005 | 5938 | 1088 | 7049 | 982 | -326 | 899 |
|  | Mean | 7737 | 1257 | 6984 | 1190 | 6619 | 1361 | 7971 | 1226 | 6178 | 1041 | 5828 | 1192 | 9130 | 1100 | 6946 | 1035 | 5809 | 1151 | 6773 | 1174 | -458 | 704 |
